# Etymologia: Penicillin

**DOI:** 10.3201/eid2501.ET2501

**Published:** 2019-01

**Authors:** Ronnie Henry

**Keywords:** penicillin, drug, Alexander Fleming, Penicillium, mold, fungi, antimicrobial resistance, Staphylococcus, bacterial infections, Howard Florey, Ernst Chain

## Penicillin [penʺĭ-silʹin]

In 1928, while studying *Staphylococcus* bacteria at Saint Mary’s Hospital in London, Alexander Fleming noticed that one of his petri dishes was contaminated with mold, which was causing the bacteria near it to lyse. Because the mold was identified as belonging to the genus *Penicillium* (Latin for “brush,” referring to the chains of conidia that resemble a paintbrush or broom), Fleming named the antibacterial substance penicillin (Figure).

**Figure:**
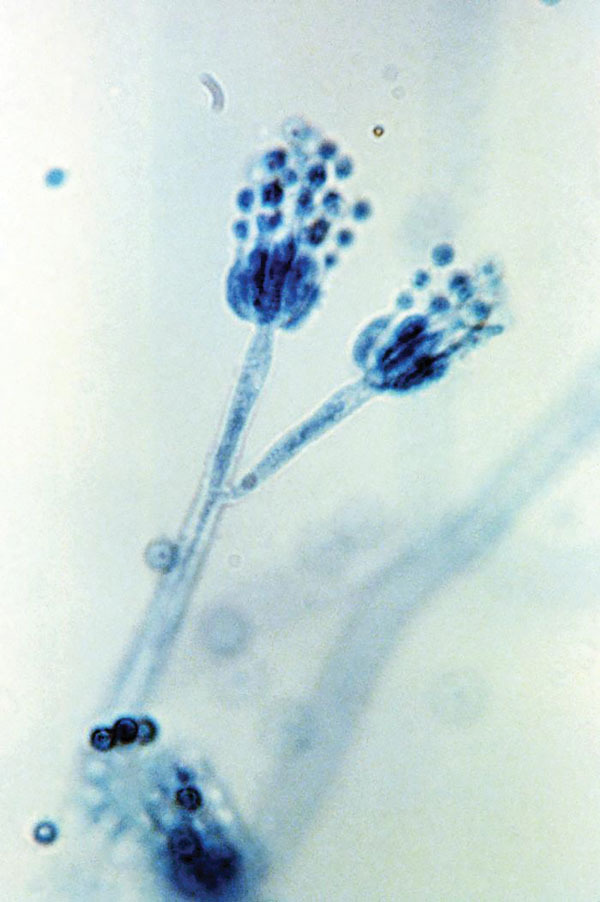
Two conidiophores of *Penicillium frequentans* fungi, also known as *P. glabrum*. The conidiophore is the stalked structure whose distal end produces asexual spores (conidia) by budding. Original magnification x1,200. Photo: CDC/Lucille Georg/1971.

Among the earliest known clinical uses of penicillin was by Cecil George Paine, a pathologist at the Sheffield Royal Infirmary, who successfully used it in 1930 to treat gonococcal conjunctivitis in neonates. Thereafter, the therapeutic potential of penicillin went largely unexplored until 1940, when a team of researchers headed by Howard Florey and Ernst Chain showed that it produced dramatic improvements in mice with streptococcal infections. Penicillin was instrumental in treating infections in Allied soldiers in World War II; however, shortly thereafter, resistance became a substantial clinical problem.
